# Lactation alters the relationship between liver lipid synthesis and hepatic fat stores in the postpartum period

**DOI:** 10.1016/j.jlr.2022.100288

**Published:** 2022-09-23

**Authors:** Maria A. Ramos-Roman, Majid M. Syed-Abdul, Brian M. Casey, Jeffry R. Alger, Yu-Lun Liu, Elizabeth J. Parks

**Affiliations:** 1 Division of Endocrinology, Department of Internal Medicine, University of Texas Southwestern Medical Center, Dallas, TX, USA; 2 Department of Nutrition and Exercise Physiology, University of Missouri School of Medicine, Columbia, MO, USA; 3 Division of Maternal and Fetal Medicine, Department of Obstetrics & Gynecology, University of Alabama, Birmingham, AL, USA; 4 Advanced Imaging Research Center, University of Texas Southwestern Medical Center, Dallas, TX, USA; 5 Neurospectroscopics LLC, Sherman Oaks, CA, USA; 6 Department of Neurology, Geffen School of Medicine at UCLA, University of California, Los Angeles, Los Angeles, CA, USA; 7 Hura Imaging, Calabassas, CA, USA; 8 Department of Population and Data Sciences, University of Texas Southwestern Medical Center, Dallas, TX, USA

**Keywords:** hormones, lipogenesis, liver metabolism, mammary gland, nonesterified fatty acids, pregnancy, prolactin, triacylglycerol, VLDL, Adipo-IR, adipose tissue insulin resistance, AIRC, Advanced Imaging Research Center, DNL, de novo lipogenesis, EGP, endogenous glucose production, GDM, gestational diabetes, ^1^H-MRS, proton magnetic resonance spectroscopy, HOMA-IR, homeostatic model assessment of insulin resistance, IHTG, intrahepatic triacylglycerols, NAFLD, nonalcoholic fatty liver disease, RaFFA, rate of appearance of nonesterified fatty acids

## Abstract

In mothers who are nursing their infants, increased clearance of plasma metabolites into the mammary gland may reduce ectopic lipid in the liver. No study to date has investigated the role of lactation on liver lipid synthesis in humans, and we hypothesized that lactation would modify fatty acid and glucose handling to support liver metabolism in a manner synchronized with the demands of milk production. Lactating (n = 18) and formula-feeding women (n = 10) underwent metabolic testing at 6-week postpartum to determine whether lactation modified intrahepatic triacylglycerols (IHTGs), measured by proton magnetic resonance spectroscopy. Subjects ingested oral deuterated water to measure fractional de novo lipogenesis (DNL) in VLDL-TG during fasting and during an isotope-labeled clamp at an insulin infusion rate of 10 mU/m^2^/min. Compared with formula-feeding women, we found that lactating women exhibited lower plasma VLDL-TG concentrations, similar IHTG content and similar contribution of DNL to total VLDL-TG production. These findings suggest that lactation lowers plasma VLDL-TG concentrations for reasons that are unrelated to IHTG and DNL. Surprisingly, we determined that the rate of appearance of nonesterified fatty acids was not related to IHTG in either group, and the expected positive association between DNL and IHTG was only significant in formula-feeding women. Further, in lactating women only, the higher the prolactin concentration, the lower the IHTG, while greater DNL strongly associated with elevations in VLDL-TG. In conclusion, we suggest that future studies should investigate the role of lactation and prolactin in liver lipid secretion and metabolism.

During lactation, milk production dominates maternal metabolism and requires cooperation between neuro-endocrine systems and metabolic tissues (1, 2). This cooperation appears to restrict nutrient utilization in nonmammary tissues to basal requirements (3) and to allow for bursts in nutrient utilization in insulin-dependent tissues after meals. Such lactational changes associated with milk production could serve to spare the liver from accumulation of newly-made lipids. In these processes, prolactin, progesterone, and insulin are three hormones of interest because of their potential effects on hepatic lipogenesis and intrahepatic triacylglycerol (IHTG). In rodents, prolactin has been shown to decrease hepatic lipogenesis and thus reduce the availability of substrates for lipid synthesis in the maternal liver (4), while in humans, the influence of prolactin on the lipogenic pathway in vivo in the liver has not been studied before. However, in human liver samples from obese subjects with and without nonalcoholic fatty liver disease (NAFLD), those with NAFLD exhibited lower serum concentrations of prolactin (5).

In the general population, hepatic steatosis is a harbinger of cardiometabolic risk (6, 7). Higher levels of prolactin associate with a reduced risk for NAFLD in men and in women (8). Further, in women, longer duration of lactation and repeated exposure to lactation during subsequent pregnancies prolong the maternal exposure to physiologic hyperprolactinemia, which is associated with a lower prevalence of hepatic steatosis during perimenopause (9). In support of these clinical observations, studies in rodent models agree with a role for lactation to reduce liver stores of triacylglycerols (TGs) (10).

Progesterone is produced by the placenta during pregnancy and by the ovaries in nonpregnant, nonpostpartum women. Importantly, progesterone stays at low levels for a variable period after delivery during lactational amenorrhea (11). In rodents, high prolactin and low progesterone have been shown to decrease hepatic lipogenesis (4, 12), increase the number of insulin receptors and the rate of fatty acid synthesis in mammary tissue (13, 14), and decrease the number of insulin receptors and the rate of fatty acid synthesis in adipose tissue (13, 14). Finally, concerning insulin, the increased clearance rate of insulin by the lactating mammary gland is an important driver of low plasma insulin concentrations (15), and low insulin concentrations may reduce IHTG by decreasing hepatic lipogenesis and increasing fat oxidation (16).

The aim of the current study was to investigate the relationships between metabolic variables and IHTG in women during active lactation, a state of physiologic hyperprolactinemia. This was accomplished by measuring substrate availability (fatty acids and glucose) and oxidation, adipose lipolysis, hepatic lipogenesis, and VLDL-TG during basal and insulin-stimulated conditions in lactating and formula-feeding women at 6-week postpartum. Understanding these early effects is important because lasting modifications in nutrient utilization after weaning could link the intensity and duration of breastfeeding with lower hepatic steatosis and lower maternal cardiometabolic risk.

## Materials and methods

### Study design

The 28 postpartum women consenting to participate in the current study were presented in an earlier report that described the effects of lactation on adipose and liver metabolic responses at 6-week postpartum (3). That earlier report focused on the rate of appearance of nonesterified fatty acids (RaFFA), endogenous glucose production (EGP), rate of glucose utilization (Rd glucose), and IHTG in 12 lactating and 6 formula-feeding women. The other 10 subjects in the earlier report only contributed data for the insulin concentration for half-maximal suppression of RaFFA. The present report focuses on the impact of lactation on hepatic de novo lipogenesis (DNL) and liver lipids measured at the same time in all 28 postpartum subjects participating in the earlier study. Specifically, the subjects ingested deuterated water and were studied in the basal state, as well as during a hyperinsulinemic-euglycemic clamp with stable isotopes that focused on adipose fatty acid flux at a single insulin infusion rate of 10 mU/m^2^/min. This low insulin infusion rate serves to assess the suppression of both lipolysis and EGP in nonpregnant and nonpostpartum humans because of the higher insulin sensitivity of adipose tissue and liver versus skeletal muscle (17). Data were collected between 5- and 8-week postpartum. Participants were classified as lactating or formula-feeding women based on their choice of infant-feeding regimen and the groups were compared in a cross-sectional analysis.

### Subjects, inclusion, and exclusion criteria

As described previously (3), potential participants were approached at a scheduled prenatal visit during the third trimester of pregnancy and/or at the scheduled postpartum visit between 2 and 5 weeks from delivery. Inclusion criteria were age 21–49 years, confirmed normal glucose tolerance or diet-treated gestational diabetes (GDM) during the third trimester of pregnancy, singleton delivery at term, BMI at 2-week postpartum between 25 and 35 kg/m^2^, and intention to either feed mostly breast milk (≤6 oz of formula per day) or formula only. Exclusion criteria were history of pregestational diabetes, drug-treated GDM, preeclampsia, hormonal contraception or intrauterine device, pregnancy, use of medications that interfere with prolactin release or nutrient metabolism, postpartum depression, contraindications for MRI, uncontrolled hypothyroidism, and liver or kidney disease. The research protocol was approved by the Institutional Review Board at UT Southwestern Medical Center (number STU-092010-071), and the study was conducted according to the principles expressed in the Declaration of Helsinki.

### Study visits

Subjects fasted 12–14 h before each visit and visit 1 included screening tests, 2-h OGTT, and body composition measured by dual-energy X-ray absorptiometry. Lactating women pumped breast milk simultaneously from both breasts for 20 min using an electric, hospital-grade dual breast pump (Ameda®) between 10 and 30 min before oral ingestion of 75 g of dextrose. This approach allowed standardized stimulation of prolactin release relative to metabolic measurements considering that prolactin levels fluctuate relative to time from nursing, peaking around 30 min from initiation of lactation (18). Visit 2 consisted of ingestion of deuterated water, hormonal measurements in the fasting state, indirect calorimetry to measure substrate oxidation, and a low-dose, hyperinsulinemic-euglycemic clamp. Visit 3 involved proton magnetic resonance spectroscopy (^1^H-MRS) of the liver using a 3T magnet at 9:00 AM after a 12-h fast. In lactating women, the scan was done 30 min after the start of a 20-min session of milk pumping. Visit 1 took place at the Advanced Imaging Research Center (AIRC) or at the Clinical Research Unit at UT Southwestern. Visits 2 and 3 took place at the AIRC.

For visit 2, participants arrived at the AIRC at UT Southwestern at 8:00 AM for the clamp procedure. They had previously ingested three doses of 70% deuterated water overnight to quantitate hepatic DNL (19, 20). An antecubital IV line in one arm was used to administer isotope and clamp infusates. A contralateral IV line was placed between the wrist and the antecubital region for blood draws and was covered with a heating pad between draws. Infusions with stable isotopes were started in the fasting state (120 min before initiation of the low-dose, hyperinsulinemic-euglycemic clamp) and continued during the 2 h of the clamp. Lactating women pumped breast milk at the start of the basal fasting period (0–20 min) and at the start of the low-dose clamp (120–140 min). The details of the clamp and calorimetry have been published previously (3). Hormone concentrations of prolactin, progesterone, estradiol, leptin, and total adiponectin were measured during the basal period at 25 and 30 min.

### Stable isotopes, calculations, and statistical analysis

Stable isotopes were purchased from Cambridge Isotope Laboratories, Inc (Andover, MA). Oral deuterated water was administered to assess hepatic DNL. A total dose of 70% deuterated water was calculated as 5 g per kg body water, with body water as 50% of total body weight in kg. Participants ingested a third of the total dose at each of three times (10 PM, 2 AM, and 6 AM) (19, 20). The mean (SD) atom % enrichments in plasma for deuterium were 0.0156 ± 0.0008% and 0.3632 ± 0.0459% before and after deuterium ingestion, respectively. The fraction of palmitate in VLDL-TG made from liver DNL was calculated using mass isotopomer distribution analysis (21). This fraction reflects intrahepatic lipid synthesis irrespective of the concentration of VLDL in plasma (22, 23, 24, 25). Absolute lipogenesis was calculated by multiplying fractional VLDL-TG DNL by the fasting VLDL-TG concentration, which reflects both hepatic lipid synthesis and VLDL turnover rate (19, 20). A primed-continuous infusion of [U-^13^C_6_]-glucose (20 μmol/kg over 1 min, followed by 0.4 μmol/kg/min) and a continuous infusion of K^+^[1,2,3,4-^13^C_4_] palmitate (8.05 μg/kg/min) complexed to albumin (ratio of 2 mol fatty acid to 1 mol albumin) were administered to quantitate EGP and RaFFA, respectively (23).

### Assays and equipment

Glucose concentrations were measured by using a glucose analyzer (YSI Model 2300-D Stat Plus; Yellow Springs, OH). Insulin was analyzed by ELISA (ultrasensitive ALPCO kit 80-INSHUU-E01.1). The detection limit of the assay was 0.135 μU/ml. The intra-assay coefficient of variation ranged 2.3%–6.9% (mean 4.2%). Leptin (kit HADK2MAG-61K; Millipore) and total adiponectin (kit 80-ADPHU-E01; ALPCO) were analyzed by ELISA and FFA by a colorimetric method (#991-34891; Wako). Prolactin, progesterone, and estradiol concentrations were analyzed by Quest clinical laboratories. To measure IHTG, sagittal, axial, and coronal images through the liver were obtained and these were used to position a 27 cm^3^ ^1^H-MRS volume of interest entirely within the liver (26). ^1^H-MRS data were acquired using the Philips STEAM single voxel MRS pulse sequence (TR 1,600 ms, TE 14 ms) on a 3T MRI system. Water suppressed and water not suppressed spectra were obtained. Data were processed using custom-written software (SVFit2016) to determine integrated real intensities of water and triacylglycerol signals, which were used to compute IHTG. An IHTG value >5.5% was used to indicate the presence of hepatic steatosis (27). Food intake was measured using a validated food frequency questionnaire (Block 2005, Nutritionquest.com) (28).

### Insulin sensitivity and insulin secretion indices

We used previously published equations to calculate the homeostatic model assessment of insulin resistance (HOMA-IR) and adipose tissue insulin resistance (Adipo-IR) (29, 30). The Insulin Secretion-Sensitivity Index-2, a measure analogous to the disposition index obtained from the IVGTT, was calculated as the product of (AUC_insulin_/AUC_glucose_) and Matsuda Index (31, 32). The Stumvoll first phase insulin secretion index was calculated as 1,194 + (4.724 × Ins_0_) − (117.0 × Gluc_60_) + (1.414 × Ins_60_) (33). The Stumvoll second phase index was calculated as 295 + (0.349 × Ins_60_) − (25.72 × Gluc_60_) + (1.107 × Ins_0_) (33). The Stumvoll indices use glucose in mmol/l and insulin in pmol/l. We converted the final insulin results from pmol/l to μU/ml.

### Statistical analysis

All statistical analyses are presented as mean ± SD. Two-group comparisons were made using Mann-Whitney tests. Two-way ANOVA was conducted to simultaneously evaluate responses to insulin infusion rates (0, 10 mU/m^2^/min) and feeding method using results from the end of the basal period (95, 105, and 115 min) and results from the end of the low-dose insulin infusion rate (215, 225, and 235 min). The figures reporting Spearman correlation analyses were used to evaluate associations between IHTG, prolactin, and lipogenesis. A two-sided *P*-value ≤0.05 was considered statistically significant. *P*-values were not adjusted for multiple testing. Statistical analysis was performed with GraphPad Prism (Version 9.3.0, San Diego, CA) and StatView® (Version 5.0.1, 2008).

We also used multivariable regression analysis to investigate whether prolactin could predict other outcomes of interest after adjusting for the following two potential confounders: prepregnancy weight and percent body fat at 6-week postpartum. The outcomes of interest were fasting hormones (insulin, leptin, total adiponectin, progesterone), HOMA-IR, Matsuda Index (31), first phase Stumvoll (33), second phase Stumvoll (33), Adipo-IR (34), HDLc, fasting VLDL-TG, fasting RaFFA, fasting percentage of VLDL-TG from DNL, amount of glucose infused during the clamp in grams, and IHTG. The logarithmic transformation was applied to the outcomes if the assumption of normality for the residuals was not satisfied. We also used multivariable regression to evaluate the known predictors of IHTG.

## Results

Participant characteristics are shown in Table 1. Lactating and formula-feeding women were not different with respect to age, parity, history of GDM, IHTG, or body composition. Likewise, no differences were found between the groups for maternal weight (before pregnancy, at term and at 6-week postpartum), gestational age at delivery, infant birth weight, waist-to-hip ratio at 6-week postpartum, or family history of type 2 diabetes (data not shown). Fasting EGP in absolute units and HDLc concentration were 30% and 18% greater, respectively, in lactating women. Although total plasma TGs were not different between the groups, VLDL-TG was 56% lower in lactating women (*P* = 0.020), which at a mean concentration of 0.4 mmol/l (35 mg/dl) was the lowest ever observed in our past research. The groups were not different in their HbA_1c_ levels or fasting glucose concentrations but fasting insulin concentrations were 42% lower in the lactating women (*P* = 0.027). This led to low values for HOMA-IR and Adipo-IR, and most indices in which fasting insulin concentration strongly influences the calculation. Compared with formula-feeding women, the lactating women exhibited 43% lower insulin secretion with a first phase Stumvoll index (*P* = 0.007) and 38% lower insulin secretion with a second phase Stumvoll index (*P* = 0.014). Insulin sensitivity was not statistically different between the groups by Matsuda Index.

**Table 1:** Characteristics of postpartum subjects

Anthropometric and Metabolic Characteristics	Lactating versus Formula Groups		
	Lactating (n = 18)	Formula (n = 10)	*P* Value
Age (years)	34 ± 4	34 ± 4	0.859
Parity	3.1 ± 0.8	2.7 ± 1.3	0.640
History of GDM in last pregnancy (%)	67	40	0.243
Exclusive lactation (%)	72	0	<0.001
BMI at 6-week postpartum (kg/m^2^)	29.8 ± 4.5	30.6 ± 2.0	0.356
Intrahepatic lipid (%)	9.9 ± 5.7	10.7 ± 6.0	0.860
Body composition by DEXA			
Fat mass (kg)	29.9 ± 9.4	29.1 ± 4.0	0.532
Fat-free mass (kg)	42.9 ± 6.1	42.6 ± 5.2	0.878
HDL cholesterol (mmol/l)	1.3 ± 0.3	1.1 ± 0.2	0.020
Total plasma TG (mmol/l)	1.4 ± 0.6	2.0 ± 1.1	0.133
VLDL-TG (mmol/l)	0.4 ± 0.3	0.9 ± 0.8	0.020
FFA (mmol/l)	0.60 ± 0.14	0.59 ± 0.13	0.768
HbA_1c_ (%)	5.7 ± 0.3	5.6 ± 0.3	0.104
Fasting glucose (mg/dl)	84 ± 8	86 ± 5	0.768
Fasting insulin (μU/ml)	2.6 ± 2.1	4.5 ± 2.4	0.027
HOMA-IR	1.0 ± 1.0	1.5 ± 0.9	0.027
Adipo-IR	2.7 ± 3.3	4.3 ± 2.6	0.011
Matsuda index	11.9 ± 9.8	6.9 ± 4.4	0.133
First-phase Stumvoll (μU/ml)	97 ± 61	170 ± 82	0.007
Second-phase Stumvoll (μU/ml)	28 ± 15	45 ± 20	0.014
Insulin secretion sensitivity index 2	2.32 ± 1.55	2.34 ± 1.04	0.471
Fasting EGP (mg/kg FFM/min)	4.3 ± 0.9	3.3 ± 0.5	0.002
Prolactin (ng/ml)	242 ± 138	14 ± 9	<0.001
Total Adiponectin (μg/ml)	4.1 ± 1.2	3.9 ± 1.0	0.588
Leptin (ng/ml)	5.1 ± 4.0	6.8 ± 2.5	0.031
Estradiol (pg/ml)	29 ± 17	75 ± 29	<0.001
Progesterone (ng/ml)	0.49 ± 0.01	1.52 ± 2.20	0.028
Fasting RQ^a^	0.82 ± 0.08	0.85 ± 0.04	0.122
Clamp RQ^a^	0.85 ± 0.07	0.89 ± 0.05	0.136

Adipo-IR, adipose tissue insulin resistance; GDM, gestational diabetes; HOMA-IR, homeostatic model of insulin resistance; RQ, respiratory quotient; TGs, triacylglycerols.

All data are mean ± SD. Subjects were women at 6-week postpartum who were either lactating or feeding their infants with formula. Mann-Whitney tests were used for unpaired cross-sectional analyses.

a A two-way ANOVA was also used on RQ because this variable was measured under basal and insulin-stimulated conditions (feeding group *P* = 0.145, time *P* = 0.003, interaction *P* = 0.408).

As expected, prolactin concentrations in the lactating women were significantly elevated and a wide variability in prolactin concentrations was observed in this group (Table 1). No differences were observed between the groups for plasma FFA or total adiponectin concentrations. Plasma leptin was 25% lower in lactating women resulting in an adiponectin/leptin ratio that was more than 2-fold higher in the lactating women. Estradiol and progesterone were 61% and 68% lower in lactating women, respectively. Whole-body fasting substrate oxidation calculated from the respiratory quotient suggested greater fat oxidation in lactating women. However, the group differences did not reach statistical significance. During the clamp, the respiratory quotient rose in both groups, indicating a stimulation of glucose oxidation.

**Figure 1:**
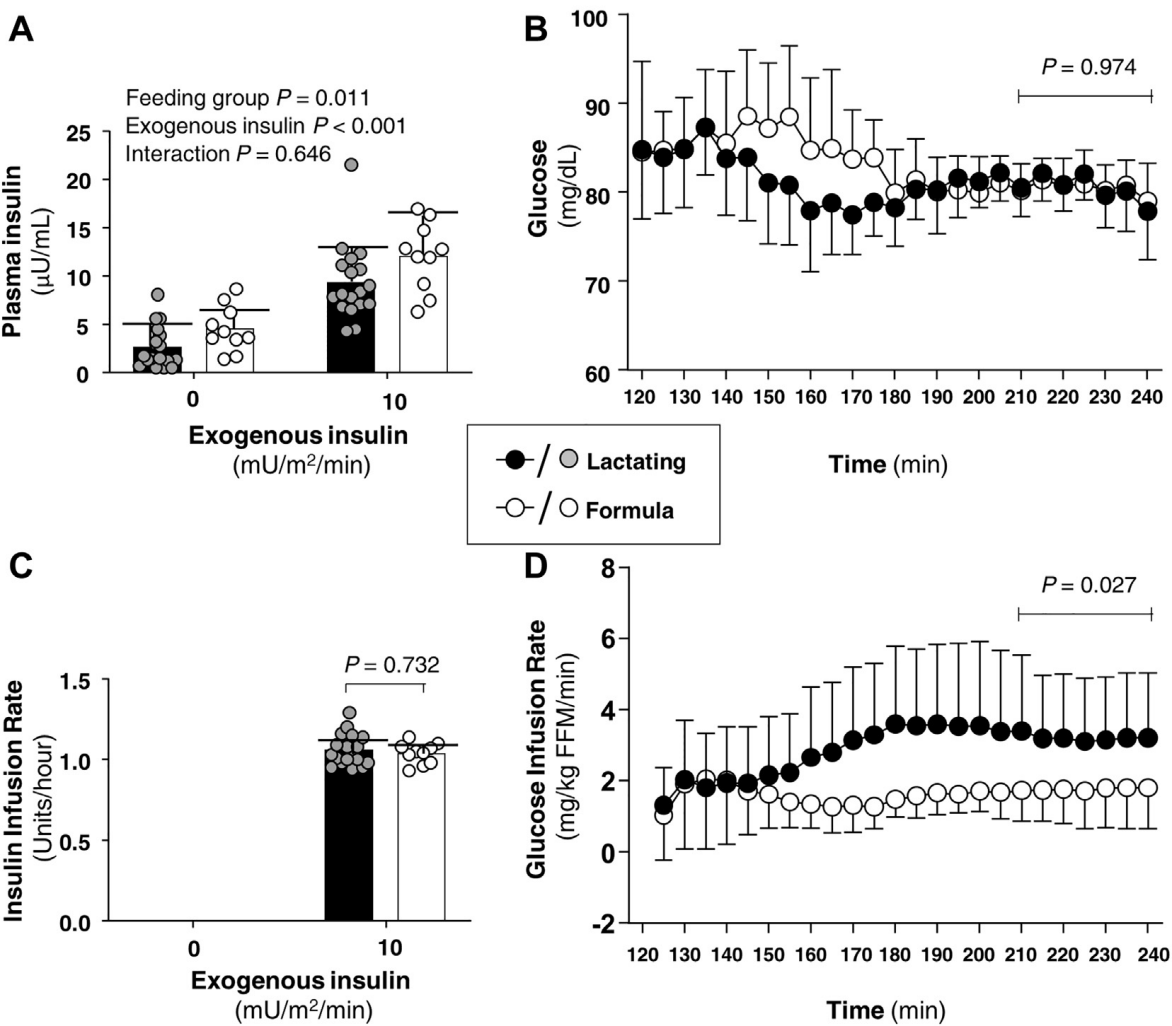
Insulin and glucose concentration immediately before and during an insulin infusion rate of 10 mU/m^2^/min and glucose infusion rate during insulin administration. A: Plasma insulin concentration at baseline and during insulin infusion. Baseline insulin concentrations were averaged for each postpartum woman using measurements from 95, 105, 115 min (before insulin) and from 215, 225, 235 min (end of insulin exposure at this rate). B: Glucose concentration at baseline (t = 120 min) immediately before insulin administration and during a 120-min clamp (t = 120–240 min) using an insulin infusion rate of 10 mU/m^2^/min. C: Units per hour of insulin infused during an insulin infusion rate of 10 mU/m^2^/min. D: Glucose infusion rate during the clamp. Data are mean ± SD. Filled bars and filled circles represent lactating women. Open bars and open circles represent formula-feeding women.

As shown in Fig. 1, the fasting insulin was lower in the lactating group. At an insulin infusion rate of 10 mU/m^2^/min, the amount of insulin infused and the glucose concentration during the last 30 min of the clamp were similar between the groups. At an insulin infusion rate of 10 mU/m^2^/min, the lactating group required a higher glucose infusion rate at a lower plasma insulin during the last 30 min of the insulin infusion.

**Figure 2:**
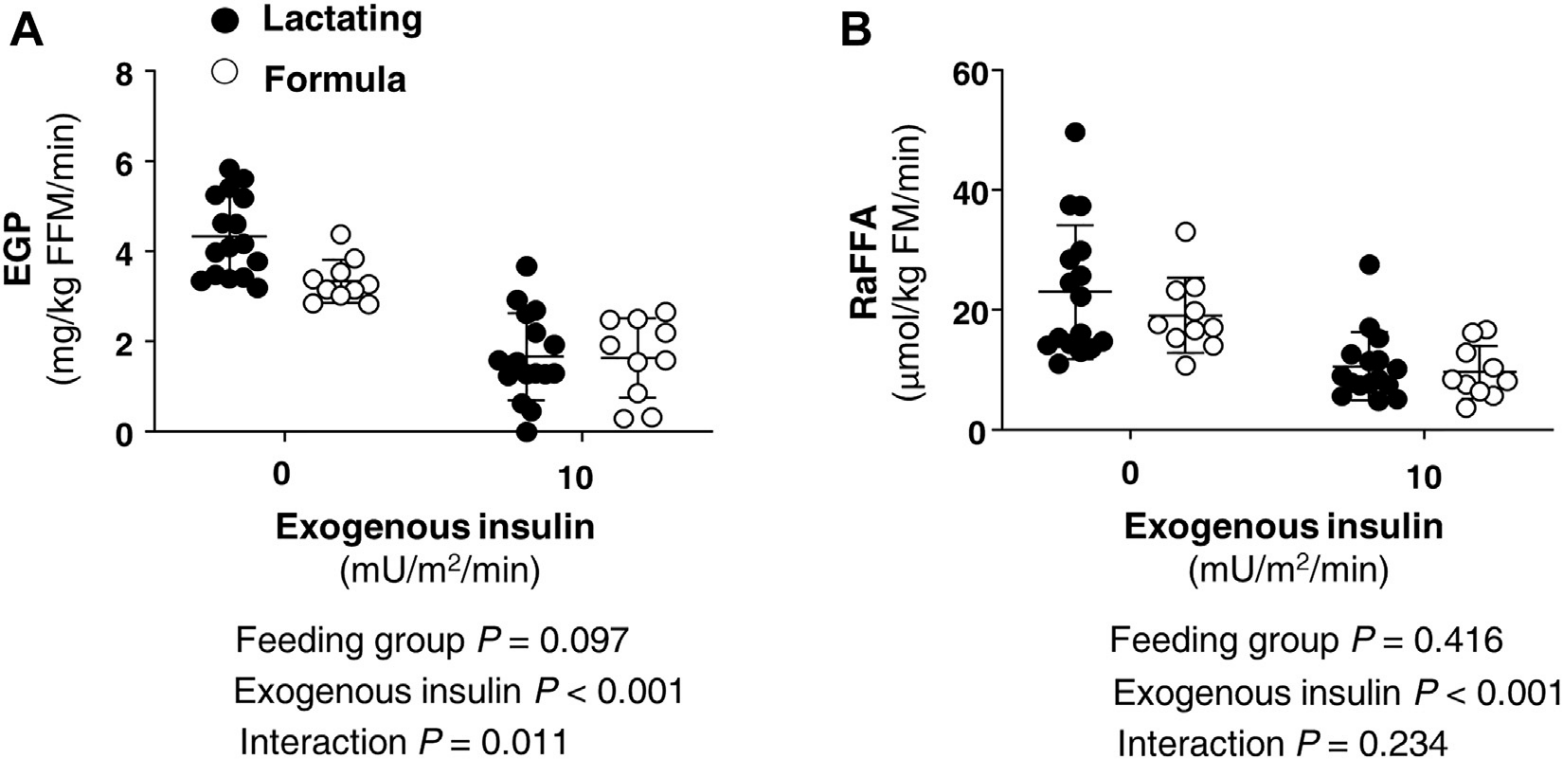
Effect of lactation on glucose production and FFA turnover. Data are mean ± SD and demonstrate the response of (A) endogenous glucose production (EGP) and (B) the rate of appearance of nonesterified fatty acid (RaFFA) during baseline (fasting state before exogenous insulin) and during the clamp with a low insulin infusion rate (10 mU/m^2^/min) for lactating (n = 18) and formula-feeding (n = 10) women. Measurements in the fasting state are the average for each postpartum woman using measurements from 95, 105, 115 min (before insulin) and from 215, 225, 235 min (end of insulin exposure at this rate). Interactions between feeding group (lactating vs. formula feeding) and exogenous insulin (insulin infusion rate of 0 or 10) were assessed with repeated-measures ANOVA. Statistical analyses are shown below each respective figure panel.

**Figure 3:**
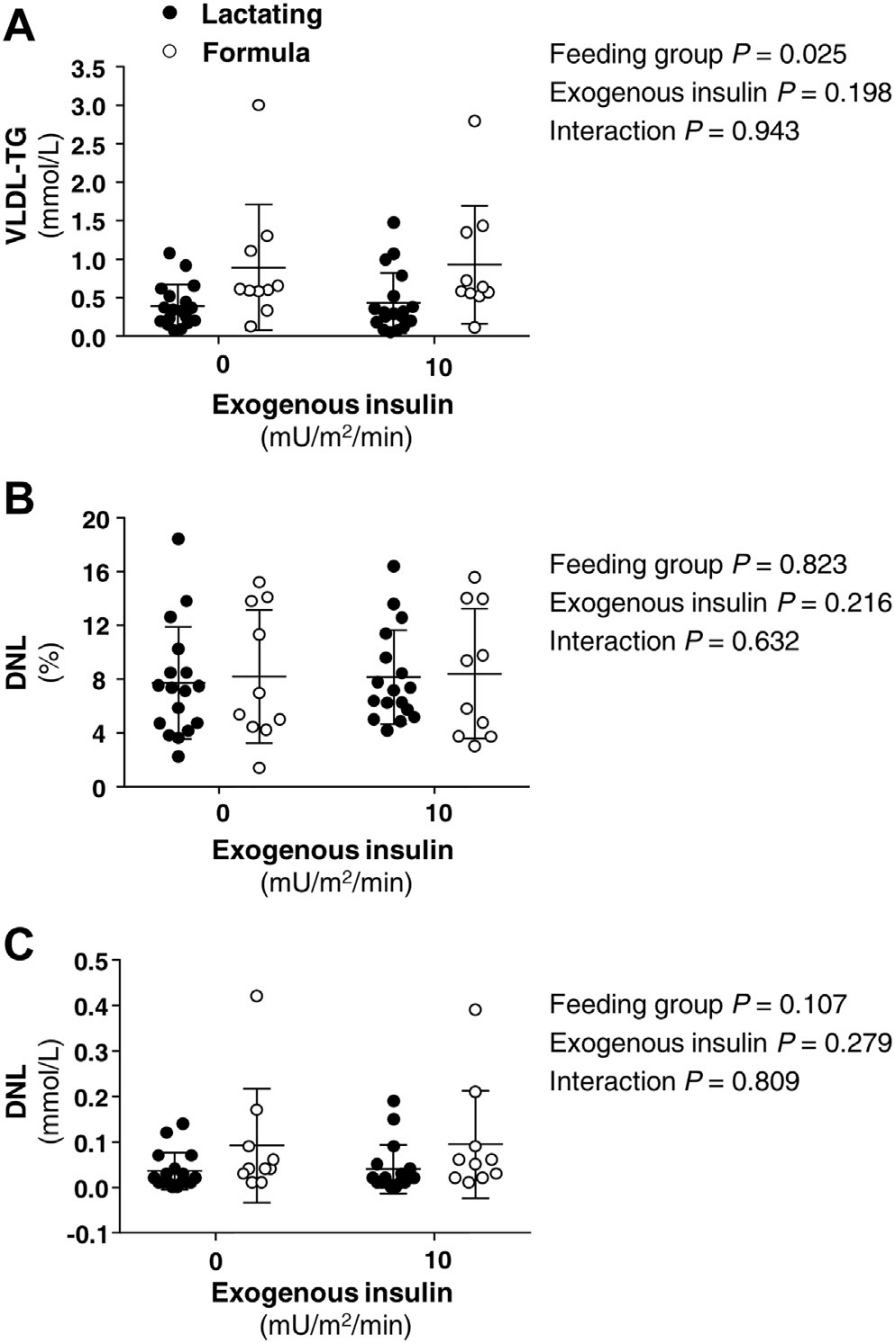
Effect of lactation and a hyperinsulinemic clamp on VLDL-TG and de novo lipogenesis. Data are mean ± SD and demonstrate the concentrations of plasma TG carried in very low-density lipoproteins (VLDL-TG in panel A), the fractional de novo lipogenesis in VLDL-TG (DNL %, panel B), and the absolute DNL (panel C) during fasting and during insulin infusion (10 mU/m^2^/min). Timing of the infusion is presented for the fasting state (95, 105, 115 min) and at the end of the insulin infusion (215, 225, and 235 min). Interactions between feeding group (lactating vs. formula feeding) and exogenous insulin (insulin infusion rate of 0 or 10) were assessed with repeated-measures ANOVA. Statistical analyses are shown next to each respective figure panel. DNL, de novo lipogenesis.

As shown in Fig. 2A, absolute EGP (not normalized for insulin) tended to be elevated by 30% in lactating women in the fasting state and the suppression during the low insulin infusion rate was significantly greater (feeding group by time interaction *P* = 0.011). Regarding lipolysis, the clamp suppressed the RaFFA in both groups equally (52% suppression in lactating and 51% in formula-feeding women, Fig. 2B). VLDL-TG concentrations were not affected by the clamp (Fig. 3A). Fractional DNL was neither different between lactating and formula-feeding women in the fasting state nor during the insulin infusion rate of 10 mU/m^2^/min (Fig. 3B). Furthermore, no significant change in fractional DNL was observed with the higher insulin infusion rates of 20 and 40 mU/m^2^/min reported in our prior work (3) compared to the basal condition (supplemental Fig. S1). In agreement with previous studies (35), oral glucose weakly stimulates DNL when given alone (without fructose), and glucose and insulin at higher infusion rates did not significantly stimulate lipogenesis. Similar observations to those reported in Fig. 3B were made for the absolute levels of DNL (Fig. 3C).

**Figure 4:**
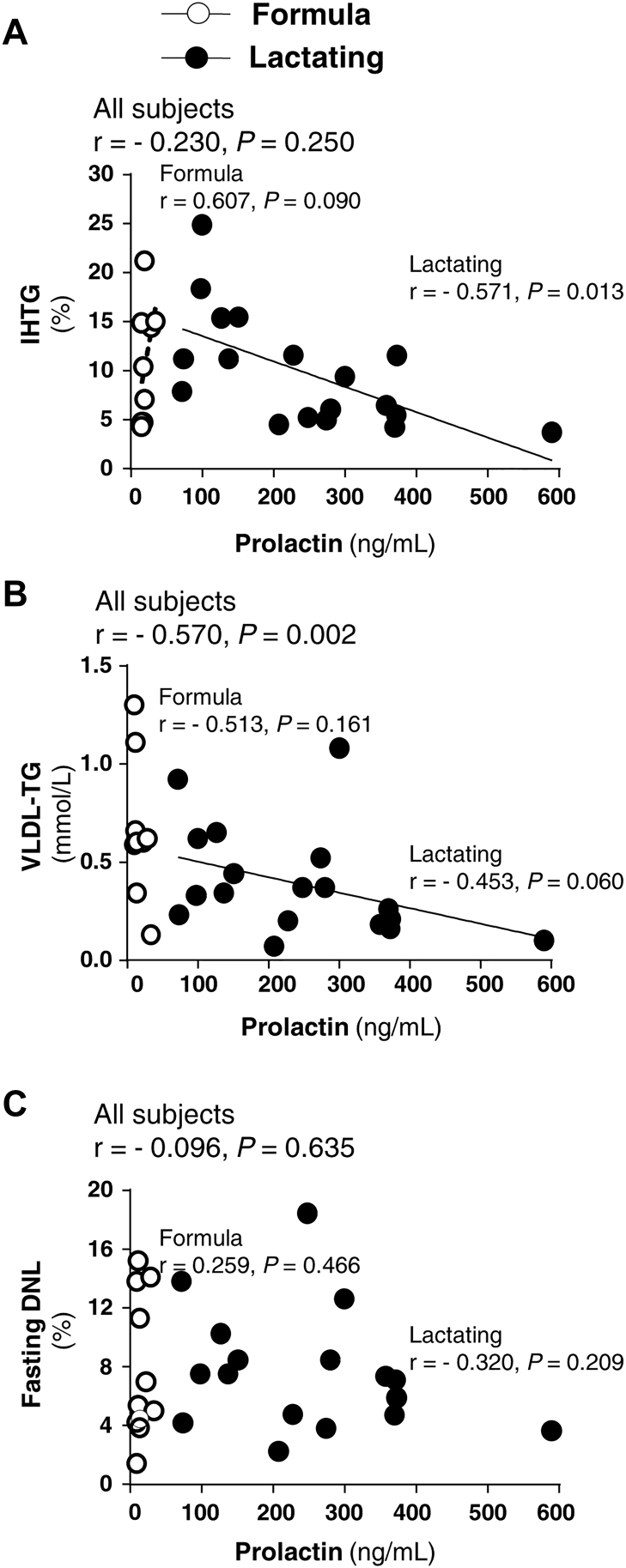
Relationships between fasting prolactin concentrations, IHTG, VLDL-TG, and fasting DNL. Spearman correlation analyses of associations between fasting prolactin concentrations and intrahepatic-TG (IHTG, panel A, lactating and formula group sample sizes n = 18 and 9, respectively), VLDL-TG concentration (panel B, n = 18 and 9), and fasting fractional de novo lipogenesis (DNL percent, panel C, n = 17 and 10). DNL, de novo lipogenesis; IHTG, intrahepatic triacylglycerol.

Variation in prolactin within the lactating group was expected (36, 37), and thus, we were able to evaluate the association between prolactin concentrations at 6-week postpartum and other variables. As shown in Fig. 4A, the higher the prolactin concentration, the lower the liver fat in lactating women by linear regression (r = −0.571, *P* = 0.013). Multiple regression analysis to test the influence of other factors commonly associated with IHTG (including prolactin, DNL, insulin, dietary carbohydrate intake (g/day and percent of total energy), postpartum percent body fat, and RaFFA) revealed that no other variable besides prolactin improved the prediction. Further, the relationship between prolactin and IHTG tended to be significant in multivariable regression analysis of data from all subjects (r = −0.547, *P* = 0.076, adjusted for prepregnancy weight and postpartum percent body fat). Figure 4B demonstrates that prolactin concentrations were inversely and significantly related to plasma VLDL-TG concentrations in an effect that appeared similar for both lactating and formula-feeding women. However, this relationship was driven statistically by the lactating group with a larger sample size. In agreement with this finding, greater prolactin predicted a decrease in fasting VLDL-TG on log scale (r = −0.586, *P* = 0.002) as analyzed by multivariable regression, again, adjusted for prepregnancy weight and postpartum percent body fat. Regarding fasting DNL (Fig. 4C), prolactin was not related to fractional lipogenesis in either lactating or formula-feeding women. Multivariable regression analysis showed that prolactin concentrations were statistically related to five additional outcomes of interest adjusted for the same two covariates. Greater prolactin predicted a decrease in fasting insulin (r = −0.517, *P* = 0.016), first and second phase Stumvoll on log scale (r = −0.447, *P* = 0.030; r = −0.428, *P* = 0.032). By contrast, greater prolactin predicted an increase in the amount of glucose infused during the clamp on log scale (r = 0.663, *P* = 0.003) and HDLc (r = 0.563, *P* = 0.012). Fasting progesterone concentration was not related to IHTG, DNL, or VLDL-TG concentrations (data not shown).

**Figure 5:**
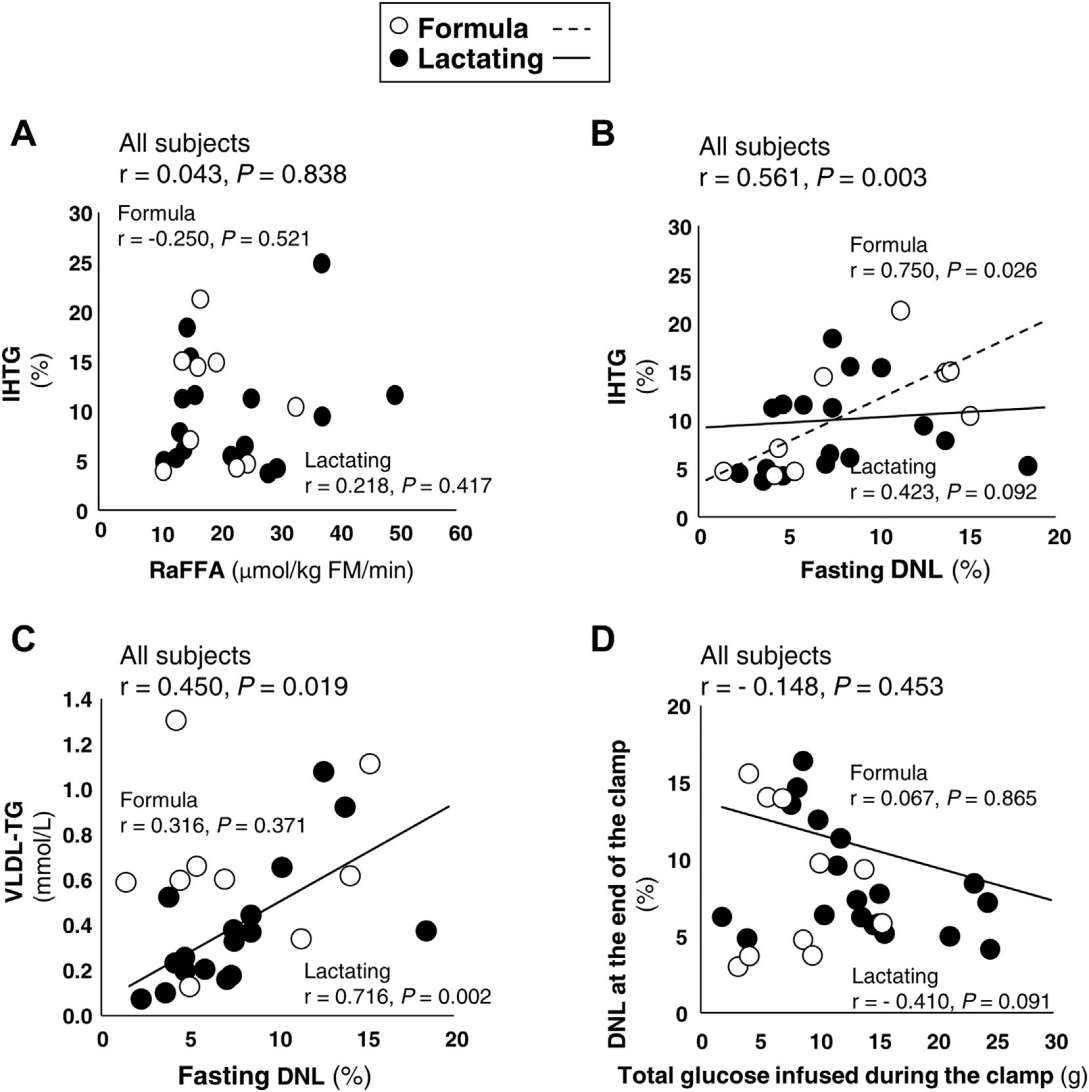
Relationships between liver fat, fasting RaFFA, DNL and VLDL-TG, and the quantity of glucose infused during the clamp. Spearman correlation analyses of associations (r, *P*-value) between the sources of liver fat from RaFFA (panel A, lactating and formula group sample sizes n = 16 and 9, respectively) and from fasting DNL (panel B, n = 17 and 9). Panel C shows the relationships between fractional DNL and VLDL-TG concentration in the fasting state (n = 17 and 9). Panel D demonstrates the lack of significant relationships between the total quantity of glucose infused during the clamp and the level of DNL at the end of the clamp (n = 18 and 10). DNL, de novo lipogenesis; RaFFA, rate of appearance of nonesterified fatty acids.

As shown in Fig. 5A, the fasting RaFFA was not related to liver fat in either group of women. In past studies, a positive relationship between fractional DNL and IHTG has been observed repeatedly (20, 22, 24, 38) and Fig. 5B demonstrates that effect here for the whole group. However, the relationship was driven by a strong effect in formula-feeding women. Surprisingly, DNL and IHTG were weakly related in lactating women. By contrast, in lactating women only, DNL was strongly and positively associated with greater plasma VLDL-TG concentrations, which suggested that lipogenesis may have been coupled to liver lipoprotein export in these women (Fig. 5C). Since both insulin and increased carbohydrate flux can stimulate hepatic lipogenesis (25), we tested whether the clamp increased DNL acutely. For all subjects combined, the clamp increased DNL from 7.9% in the fasting state to 8.5% after the clamp’s 10 mU/m^2^/min insulin infusion without statistical significance (*P* = 0.155). Nor did higher levels of insulin as reported in our previous publication (3) affect DNL over time (supplemental Fig. S1). As shown in Fig. 5D, the absolute amount of glucose infused was not related to the level of hepatic lipogenesis in either group. If anything, in lactating women, greater glucose infusion rate tended to associate with lower DNL (Fig. 5D, *P* = 0.091) suggesting that glucose clearance to the periphery lowers glucose availability for fatty acid synthesis in the liver.

## Discussion

To our knowledge, this is the first investigation of the impact of lactation on DNL and IHTG in humans. This study compared women at 6-week postpartum who chose to breastfeed or formula-feed their infants and the formula-feeding group provided a control for the effects of the postpartum period on lipid metabolism. The groups were matched for age, parity, and several metabolic characteristics. Further, within the lactating group alone, a wide range of prolactin concentrations existed which allowed for the test of novel relationships between prolactin and DNL, plasma VLDL-TG concentrations, and IHTG. The key findings of this study were that compared with formula-feeding women, lactating women exhibited lower plasma VLDL-TG concentrations, similar IHTG content, and similar contribution of DNL to total VLDL-TG suggesting that lactation lowers plasma VLDL-TG concentrations for reasons that are unrelated to IHTG and DNL. In addition, within the group of lactating women, *1*) plasma prolactin concentrations were significantly associated with lower levels of IHTG (Fig. 4A), *2*) higher fractional DNL was not related to elevated IHTG, but rather *3*) newly-made fatty acids via the DNL pathway were found in subjects with higher VLDL-TG concentrations suggesting more efficient export of lipid from the liver. These findings are strongly supported by experimental data in lactating women (9), nonpregnant humans (5), and animals (4, 12, 39) and point to a potential role for prolactin to alter lipid handling, lowering IHTG, and promoting liver health.

### Prolactin and liver fat

The magnitude of hepatic-TG storage is the net result of liver fatty acid uptake, DNL, tissue export of TG on VLDL particles, and intracellular fatty acid oxidation (40). Various conditions predispose to fatty liver including obesity and diabetes, while countervailing physiologic forces may reduce liver fat, including weight loss from energy restriction (22), exercise (41), and hormonal changes such as increased leptin (20). Outside the setting of pregnancy and the postpartum period, increased prolactin is inversely correlated with IHTG within what has been proposed as a metabolically beneficial range of 15–100 ng/ml (42). A renewed focus on the influence of prolactin to benefit liver health by reducing IHTG has led to investigations of this hormone in both humans (5) and animals (4, 39). In a study of patients undergoing weight-loss surgery by Zhang *et al.* (5), concentrations of serum prolactin were found to be 39% lower in women with fatty liver than in women without fatty liver. Further, the expression of the prolactin receptor (*PRLR*) in liver tissue was inversely related to the expression of the fatty acid transporter *CD36*. Downregulation of hepatic *CD36* may facilitate routing of fatty acids away from the liver toward the mammary gland in lactating women. Regarding the contribution of adipose fatty acid flux to liver fat accrual, studies in men and nonlactating women have produced mixed results, some demonstrating that poor adipose suppression of FFA release by insulin is related to greater IHTG (43), while more recent studies demonstrated no effect of fatty acid uptake on the level of liver fat (44).

Lactation is characterized by low insulin concentrations relative to formula-feeding and the nonpregnant/nonpostpartum state (45). To calculate peripheral insulin sensitivity, indices that include basal insulin levels, like Adipo-IR, mathematically depend on fasting insulin concentrations. Thus, low basal insulin concentrations due to lactation lead to conclusions of improved adipose insulin sensitivity over formula-feeding and the nonpregnant/nonpostpartum state. The conclusion based on Adipo-IR observed here agrees with our previous data (3) from direct quantitation of adipose insulin sensitivity as the EC_50_ RaFFA (e.g., the concentration of insulin needed to half-maximally suppress lipolysis). The EC_50_ RaFFA was 55% higher in formula-feeding women than in lactating women. Improvement in adipose insulin sensitivity during the postpartum period relative to the nonpregnant/nonpostpartum period may be further augmented by lactation (3). This is consistent with the notion that at 6-week postpartum, formula-feeding women are closer than lactating women to their prepregnancy baseline metabolism but have not yet returned to their prepregnancy metabolic state. Although adipose insulin sensitivity is elevated at 6-week postpartum, it did not appear to be related to lower IHTG, and thus the benefit of lactation on liver fat may not be mediated directly by reduced RaFFA.

### De novo lipogenesis

Within all the postpartum women included in this analysis, the ranges of IHTG were wide [lactating women (IHTG 3.7%–24.8%) and formula-feeding women (4.3%–21.2%)]. Given the normal cutoff for fatty liver in nonpregnant, nonlactating humans of 5.5% (26, 27), it was clear that many of these subjects had excess IHTG and the levels of IHTG in the two groups were not statistically different. This was slightly surprising to us, given the literature cited above, and additional studies showing that lactating women exhibit higher basal glucose export out of the liver (EGP) and lower basal insulin concentrations (3), two characteristics that would be expected to lower IHTG (46) through reduced lipogenesis. In the study of Zhang *et al.* (5), higher liver *PRLR* expression tended to associate with lower expression of the liver fatty acid synthesis gene, acetyl-CoA carboxylase. Similar relationships were found in HepG2 cells that overexpressed the prolactin receptor (5). In the present study, a strong benefit of prolactin to lower IHTG in the lactating women was not related to suppression of hepatic fatty acid synthesis, measured directly using isotopic labeling. We tested this hypothesis based on the numerous previous observations by us that DNL is a unique indicator of fatty liver (19, 20, 22). Further, Smith *et al.* (38), have demonstrated a graded increase in DNL with increasing levels of IHTG in lean and obese subjects without NAFLD, as well as in obese subjects with NAFLD. We also explored the relationship between increased dietary carbohydrate, elevated hepatic DNL, and IHTG (22), yet here dietary carbohydrate intake, expressed as a percentage of daily energy consumed or as absolute carbohydrate intake (g/day), failed to predict DNL or IHTG (data not shown). This lack of predictors of liver fat in the lactating women contrasted with data in the formula-feeding women, in which both DNL (Fig. 5B) and higher fasting insulin concentrations (r = 0.693, *P* = 0.038) were positively associated with IHTG. Thus, the present data suggest that lactation may interrupt the known effects of metabolic parameters (insulin concentration, DNL, RaFFA) that increase IHTG.

In sum, the experimental data suggest a dominant effect of secretion of TG out of the liver in concert with efficient clearance of VLDL-TG from plasma as driving forces to decrease postpartum liver fat, two pathways that should be the focus of future research. Higher plasma prolactin levels were associated with *1*) lower IHTG, *2*) lower concentrations of plasma VLDL-TG, and *3*) higher fractional DNL fatty acids found in VLDL-TG. The higher fractional DNL reflects greater hepatic use of DNL fatty acids for VLDL-TG assembly, regardless of the total quantity of TG secreted from the liver. Women who feed breast milk for a prolonged time experience a decrease in their circulating concentrations of VLDL-TG during the period of lactation followed by normalization of the circulating VLDL-TG when lactation ends (47). In rodents, the action of LPL on circulating TG-rich lipoproteins is the primary source of fatty acids for the mammary gland (48) and thus, the lower VLDL-TG in lactating women could be attributed to an increased rate of removal of VLDL-TG from plasma. Increased VLDL clearance could also explain the increased HDLc observed in the lactating women as a result of transfer of surface remnants from VLDL catabolism (48). If this is the case, increased VLDL secretion may reduce liver-TG stores (44) resulting in an inverse relationship between IHTG and VLDL lipids in plasma (49). We speculate that prolactin may have stimulated both hepatic recycling of fatty acids into VLDL synthesis and increased lipid clearance from plasma via LPL (50). These are important hypotheses to be investigated in future studies.

## Limitations

This study had several limitations. First, inherent in the intensive metabolic protocols performed, sample sizes are limited due to the challenges of collecting data in this population. In this context, although blood TG decreased during lactation (48, 51) and insulin sensitivity indices that rely on fasting insulin give the impression that lactating women are more insulin sensitive to glucose uptake and utilization, the current project was not powered to detect differences in serum TG or the Matsuda Index. Second, increased clearance of plasma TG is based on interpretation of extensive data from the literature (50, 52, 53, 54), rather than on direct measurement of VLDL-TG clearance in these subjects. However, there is past work describing a faster return of TG concentrations to prepregnancy levels in lactating women than formula-feeding women (51). At the time of the study design, we did not know that VLDL-TG secretion might be implicated in the phenotype of the lactating women and measurement of VLDL-TG turnover would have required an additional study visit with an overnight stay and a prolonged fast. Third, the short duration of labeling with deuterated water was chosen to reduce subject burden although this duration results in lower levels of fasting DNL than longer (10 days) durations (22), an effect that would be present in all subjects. A fourth limitation relates to the complexity of hormone interactions that occur during lactation, which necessitates caution in attributing these findings to changes in prolactin alone. Future studies could be conducted during the transition from lactation to weaning to address the metabolic benefit of prolonging hyperprolactinemia through pharmacologic means after cessation of lactation on women with NAFLD identified before, during, or after pregnancy. Outside the physiological state of postpartum metabolism, the specific effects of prolactin on liver metabolism could be assessed through acute prolactin infusion studies in nonpregnant/nonpostpartum women and men, possessing varying levels of IHTG, with concomitant measurements of VLDL-TG secretion rate and TG clearance.

## Conclusions

Lactation is a process that integrates whole-body substrate flux to sustain milk production without compromising basal and postprandial metabolism in the mother. This process is under the control of the hormone prolactin in partnership with several other hormones that change in concentration according to the metabolic activity of the mammary gland and time postpartum. The present data are consistent with lactation-associated elevations in prolactin modulating nutrient partitioning to lead to improvements in whole-body TG flux and the possible effect of prolactin to route newly-made fatty acids into VLDL-TG for secretion by the liver. Future studies are needed to confirm these hypotheses. The lack of expected relationships between lactation-associated levels of insulin, RaFFA or DNL, and IHTG are important to identify in themselves and highlight that *1*) relationships between metabolic variables in the postpartum setting are clearly not accurately predicted from studies of nonpostpartum human subjects (i.e., hypotheses need to be tested directly in a lactating population) and *2*) measurements of metabolism in these women are needed to support the field in developing and refining strategies to aid postpartum women to maximize metabolic health. Regarding fatty liver in other populations, further investigations focusing on the hepatic effects of prolactin may also shed new light on mechanisms designed to reduce liver fat in men and women with metabolic diseases.

## Data availability

The data sets generated during this study are available from the corresponding author upon reasonable request. No applicable resources were generated or analyzed during the current study.

## Supplemental data

This article contains supplemental data (3).

## Conflict of interest

The authors declare that they have no conflicts of interest with the contents of this article.
