# Supplementary material for: Lactation alters the relationship between liver lipid synthesis and hepatic fat stores in the postpartum period
Source: J Lipid Res. 2022 Sep 23;63(11):100288. doi: 10.1016/j.jlr.2022.100288 (PMC9619182; doi:10.1016/j.jlr.2022.100288)
Supplement: Supplemental Figure S1 [file mmc1.docx]

**SUPPLEMENTAL DATA:**

**Lactation alters the relationship between liver lipid synthesis and hepatic fat stores**

**in the postpartum period**

Maria A. Ramos-Roman^1^, Majid M. Syed-Abdul^2^, Brian M. Casey^3^, Jeffry R. Alger^4-7^,

Yu-Lun Liu^8^, Elizabeth J. Parks^2^

^1^ Department of Internal Medicine, Division of Endocrinology, University of Texas Southwestern Medical Center, Dallas, TX

^2^Department of Nutrition and Exercise Physiology, University of Missouri School of Medicine, Columbia, MO

^3^Department of Obstetrics & Gynecology, Division of Maternal and Fetal Medicine, University of Alabama, Birmingham, AL

^4^Advanced Imaging Research Center, University of Texas Southwestern Medical Center, Dallas, TX

^5^Neurospectroscopics LLC, Sherman Oaks, CA

^6^Department of Neurology, Geffen School of Medicine at UCLA, University of California, Los Angeles, Los Angeles, CA

^7^Hura Imaging, Calabassas, CA

^8^Department of Population and Data Sciences, University of Texas Southwestern Medical Center, Dallas, TX

**Supplemental Figure S1.** Fractional DNL (DNL%) is presented under basal conditions and during one or two levels of hyperinsulinemia. (**A**) Baseline and insulin infusion rate (IIR)=10 mU/m^2^/min (n=27). (**B**) Baseline and IIR=10 and 20 mU/m^2^/min (n=18). (**C**) Baseline and IIR=10 and 40 mU/m^2^/min (n=9). The current manuscript presents available results for fractional DNL from 27 women studied in the basal state and during IIR of 10 mU/m^2^/min. Eighteen of these 27 women participated in an experimental protocol with measurements in the basal state and during IIR of 10 and 20 mU/m^2^/min (1). The remaining 9 women were studied under the experimental protocol of measurements in the basal state and during IIR of 10 and 40 mU/m^2^/min (1). Data are shown individually and as mean and SD.

**Reference**

1. Ramos-Roman, M. A., Syed-Abdul, M. M., Adams-Huet, B., Casey, B. M., Parks, E. J. (2020) Lactation versus formula feeding: Insulin, glucose, and fatty acid metabolism during the postpartum period. *Diabetes* 69, 1624-1635
